# Neutralization of Rubella Vaccine Virus and Immunodeficiency-Related Vaccine-Derived Rubella Viruses by Intravenous Immunoglobulins

**DOI:** 10.1093/infdis/jiae182

**Published:** 2024-04-09

**Authors:** Min-hsin Chen, Ludmila Perelygina, LiJuan Hao, R Suzanne Beard, Cornelia Lackner, Maria R Farcet, Michael Karbiener, Joseph Icenogle, Thomas R Kreil

**Affiliations:** Viral Vaccine Preventable Diseases Branch, National Center for Immunization & Respiratory Diseases, Centers for Disease Control and Prevention, Atlanta, Georgia, USA; Viral Vaccine Preventable Diseases Branch, National Center for Immunization & Respiratory Diseases, Centers for Disease Control and Prevention, Atlanta, Georgia, USA; Viral Vaccine Preventable Diseases Branch, National Center for Immunization & Respiratory Diseases, Centers for Disease Control and Prevention, Atlanta, Georgia, USA; Viral Vaccine Preventable Diseases Branch, National Center for Immunization & Respiratory Diseases, Centers for Disease Control and Prevention, Atlanta, Georgia, USA; Global Pathogen Safety, Takeda Manufacturing Austria AG, Vienna, Austria; Global Pathogen Safety, Takeda Manufacturing Austria AG, Vienna, Austria; Global Pathogen Safety, Takeda Manufacturing Austria AG, Vienna, Austria; Viral Vaccine Preventable Diseases Branch, National Center for Immunization & Respiratory Diseases, Centers for Disease Control and Prevention, Atlanta, Georgia, USA; Global Pathogen Safety, Takeda Manufacturing Austria AG, Vienna, Austria

**Keywords:** immunodeficiency, immunodeficiency-related rubella vaccine-derived mutants, intravenous immune globulin, virus neutralization, rubella virus

## Abstract

The association between granulomas and vaccine-derived rubella virus (VDRV) in people with primary immunodeficiencies has raised concerns about the ability of immunoglobulin preparations to neutralize VDRVs. We investigated the capacity of immunoglobulin to neutralize rubella vaccine virus and 4 VDRV strains. As expected, the rubella vaccine virus itself was potently neutralized by immunoglobulin preparations, but the VDRV isolates from patients after intrahost evolution, 2–6 times less so. Diagnosis of immune deficiencies before possible live-virus vaccination is thus of critical importance, while immunoglobulin replacement therapy can be expected to provide protection from rubella virus infection.

## Teaser

This study confirmed potent rubella virus neutralization by immunoglobulin preparations, at levels that should protect people with primary immunodeficiencies. Early diagnosis and timely immunoglobulin therapy are recommended to avoid systemic spread of rubella virus, which can persist in granulomas.

Rubella is a contagious viral infection that remains a global health burden in the postvaccination era. Following vaccine introduction in 1969 in the United States [1, chap 20] and in most European countries in the 1970s and 1980s [2], rubella incidence declined dramatically. The rubella vaccine contains live attenuated rubella virus, administered typically in combination with measles, mumps, and varicella components (measles-mumps-rubella [MMR] or MMR-varicella vaccine) [1, chap 20] and, as of 2020, is recommended by 173 of 194 World Health Organization member states [3]. In immunocompetent individuals, serious adverse events following MMR vaccine are rare [4]. However, in persons who are severely immunocompromised, MMR and MMR-varicella vaccinations are contraindicated [1, chap 20].

The first MMR dose is usually given at age 12–15 months and therefore is often administered to children with undiagnosed primary immunodeficiencies (PIDs), also known as inborn errors of immunity [5]. Rubella vaccine virus can potentially establish subclinical persistent infection and then emerge as immunodeficiency-related vaccine-derived rubella virus (iVDRV) in cutaneous and visceral granulomas in individuals with PID [5–8]. iVDRV genomes contain multiple nucleotide substitutions with respect to the RA27/3 vaccine strain [5]. For prophylaxis of infections, patients with PID are typically treated with immunoglobulin preparations produced from the plasma donations of healthy donors. As the rubella vaccine virus is a part of the widely used MMR vaccine, rubella virus–specific neutralizing antibodies (nAbs) can be expected in the donor plasma and consequentially in commercial immunoglobulin products derived from this plasma. However, available data to support the assumption that nAbs can be found in immunoglobulin products are based on antibody-binding assays [9, 10]. In addition, it is unclear whether the rubella vaccine-induced antibodies in commercial immunoglobulin preparations can neutralize iVDRV isolates carrying multiple amino acid substitutions in the viral proteins [7].

Data on rubella virus neutralization capacity of immunoglobulin preparations is critically important, as confirmation would alleviate concerns around the adequate protection of prophylactically treated patients with PID from virus exposure following interaction with rubella-infected persons. Here, we investigated the neutralization capacity of immunoglobulin for the rubella vaccine virus strain RA27/3 and 4 strains of iVDRV, isolated from patients with PID and granuloma and collected at different intervals after vaccination [5]. The results confirm the neutralization capacity of immunoglobulin preparations for vaccine-derived rubella virus and vaccine virus but also reveal potential limitations in treatment efficacy against persisting virus variants.

## METHODS

### Immunoglobulin Preparations

Rubella nAb titers were determined in 40 immunoglobulin lots from 2017, fractionated by the same manufacturing process from plasma collected in the United States (Gammagard Liquid; Baxalta US; n = 30) or in Austria, Germany, and the Czech Republic (KIOVIG; Takeda Manufacturing Austria; n = 10), the leading European countries for plasma collection (collectively designated the European Union [EU]). Plasma was obtained by different plasma collection modalities, either by plasmapheresis (ie, extracorporeal separation of plasma from blood cells, source plasma [n = 27]) or from whole-blood donations (ie, plasma obtained during generation of red blood cell concentrates, recovered [n = 13]).

### Preparations of Virus Stocks

Patient isolates CA, RI, LA, and OR were recovered from snap-frozen skin biopsy samples of 4 individuals with diagnosed PID. CA, RI, and LA patients received a single MMR dose at about 1 year of age, while the OR patient received 2 MMR doses at the ages of 1 and 5 years. The iVDRV isolates were characterized elsewhere [5]. Informed consent was obtained from the 4 patients. The preparation of rubella virus stocks from supernatants of Vero-infected monolayers is also described elsewhere [11].

### Neutralization Assay

Rubella virus nAbs were determined in 5–7 independent assays. First, 2-fold serial dilutions of immunoglobulin samples in cell culture medium were prepared, ranging from 1:200 to 1:2560 and mixed in a 1:1 ratio with each of the rubella virus strains adjusted to 200–500 focus-forming units (FFUs) per sample. After 1.5-hour incubation at 37°C, the sample/virus mixtures were added to Vero cell monolayers (American Type Culture Collection CCL81) in 96-well plates in duplicate. Following adsorption, the cells were overlaid with Dulbecco's modified Eagle medium (Invitrogen) supplemented with 1% fetal bovine serum and incubated at 37°C and 5% carbon dioxide for 2 days.

Infected cells were immunostained for rubella E1 protein, as described elsewhere [5], and foci of infection were counted using an enzyme-linked immunospot assay analyzer (Cellular Technology). The neutralization titer was expressed as the reciprocal of the immunoglobulin dilution that neutralized 50% of added virus (50% focus reduction neutralization titer [FRNT_50_]) by a dose-response regression analysis using Microsoft Excel.

### Data Analysis

The evaluation of differences in antibody titers against different rubella virus isolates was done using GraphPad Prism v8.1.1 software (GraphPad Software). Geometric means and standard deviations were calculated from individual values. Differences in titers were assessed by means of Student *t* test after log_2_ transformation for all groups of data.

## RESULTS

Immunoglobulin lots fractionated from plasma of US and EU origin (either source or recovered plasma) were screened for rubella nAbs against RA27/3 strain and 4 iVDRV strains. In general, all 40 immunoglobulin lots had neutralizing activity against all analyzed rubella virus strains, regardless of geographic origin and plasma collection modality. Higher mean neutralizing titers were seen against the RA27/3 vaccine strain (geometric mean titer, 1:2577), compared with the 4 iVDRV isolates analyzed (CA, 1:430; RI, 1:545; OR, 1:577; and LA, 1:1299). The greatest differences in terms of neutralizing capacity compared with the RA27/3 vaccine strain (range, 1:1050–1:6400) were observed for isolate CA (approximately 6-fold lower; 1:231–1:867), followed by isolates RI (approximately 5-fold lower; 1:200–1:1397), OR (approximately 5-fold lower; 1:400–1:1160), and LA (approximately 2-fold lower; 1:403–1:5760) (Table 1).

**Table 1.** Information on Rubella Strains Used for Neutralization Testing: Rubella Vaccine Strain RA27/3 and Immunodeficiency-Related Vaccine-Derived Rubella Virus Isolates From Patients With Primary Immunodeficiency^a^

Rubella Strain	Age at Initial Sampling, y	No. of MMR Vaccinations	Therapy	Amino Acid Substitutions, No. in Structural Proteins/No. in 4 Known Neutralizing B-Cell Epitopes	Neutralizing Capacity vs Vaccine Virus	gRNA/FFU Ratio
RA27/3	NA	NA	NA	NA	NA	237:1
LA	6	1	IVIG	19/0	∼2-Fold lower	624:1
OR	11	2	None	36/1	∼5-Fold lower	1476:1
RI	17	1	IMIG	31/0	∼5-Fold lower	303:1
CA	10	1	IVIG	22/3	∼6-Fold lower	200:1

Abbreviations: FFU, focus-forming unit; gRNA, genomic RNA; IMIG, intramuscular immunoglobulin; iVDRV, immunodeficiency-related vaccine-derived rubella virus; IVIG, intravenous immunoglobulin; MMR, measles-mumps-rubella vaccine; NA, not applicable.

^a^Data from Perelygina et al [5]. Real-time quantitative polymerase chain reaction for genomic RNA quantification was done as described by Chen et al [12].

**Figure 1.**
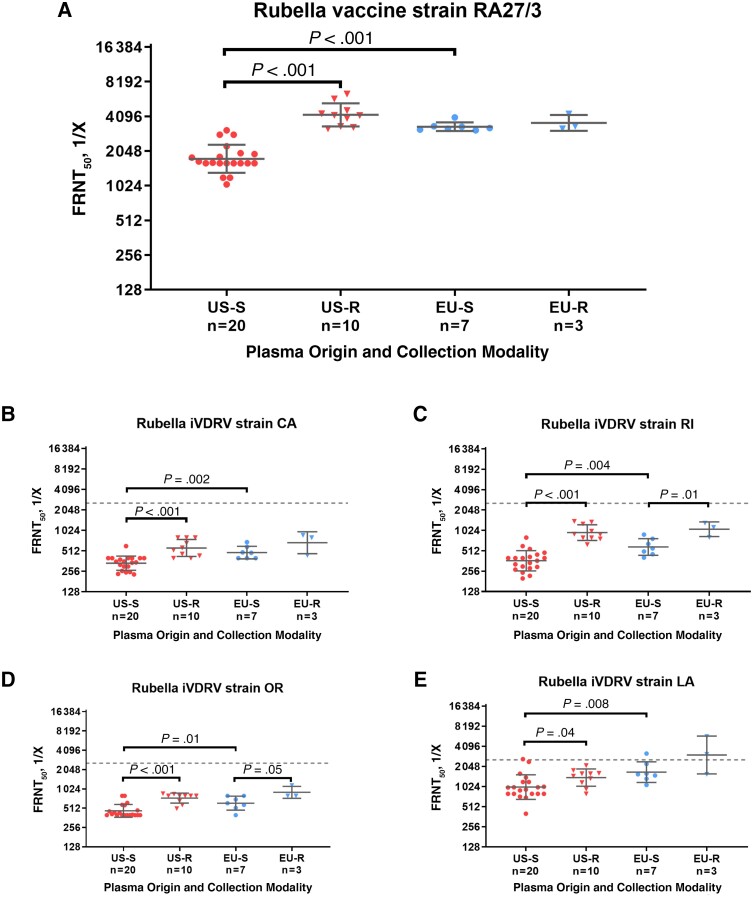
Rubella virus neutralizing antibody titers against rubella vaccine strain RA27/3 (*A*) and 4 immunodeficiency-related vaccine-derived rubella virus (iVDRV) strains (*B–E*), determined as the reciprocal dilution resulting in 50% virus neutralization (50% focus reduction neutralization titer [FRNT_50_ (1:X)]) of immunoglobulin lots, plotted according to plasma geographic origin and the collection modality used to manufacture the respective lots, which include source plasma collected in the United States (US-S) or the European Union (EU-S) and recovered plasma collected in the United States (US-R) or the European Union (EU-R). Dashed lines represent the geometric mean values of neutralizing antibody titers for all immunoglobulin lots against rubella vaccine strain RA27/3. Individual values are overlaid by geometric means with standard deviations; differences in titers were assessed using Student *t* test after log_2_ transformation. *P* values are shown for statistically significant differences, with differences considered significant at *P* < .05.

Immunoglobulin lots manufactured from source plasma collected in the United States displayed a mean rubella vaccine virus neutralization titer of approximately 1:1750 (Figure 1). These titers were significantly lower (*P* < .001) than those of immunoglobulin produced from US recovered plasma, with a mean of 1:4200. There was minor variability among individual values (range, 1:3200–1:6400), which is probably less relevant with respect to biological activity. These titers were similar to EU plasma-derived immunoglobulin, with means of 1:3500 for immunoglobulin lots from both source and recovered plasma (Figure 1). The nAb titer profile against the iVDRV isolates exhibited the same reactivity pattern, that is, markedly enhanced levels in immunoglobulin manufactured from US recovered, EU source, and EU recovered plasma compared with US source plasma-derived product (Figure 1). Within the same geography, nAb levels were higher for immunoglobulin lots derived from recovered versus source plasma (Figure 1).

To determine whether similar amounts of total viral particles were used in neutralization assays, a particle-to-FFU ratio was determined for the viral stocks. Counting viral particles in rubella virus stocks by means of electron microscopy is problematic due to low virus titers. Instead, counts of viral RNA genomes were determined by means of real-time quantitative polymerase chain reaction (Table 1) and used to represent total particle counts. Rubella virus does not induce a cytopathic effect on Vero cells, and unpackaged viral genomic RNA thus remains cell bound and does not markedly contribute to genome counts in the culture media used for virus stock preparation. Except for isolate OR, all virus stocks presented similar genome-to-FFU ratios, indicating that the levels of nAbs in the immunoglobulin preparations were measured with similar amounts of virus particles.

## DISCUSSION

In general, in high-income countries, rubella vaccination coverage is above 90% [3]. In Europe, however, the coverage varies between different regions and populations across EU member states [3]. Potent rubella virus nAbs were detected in all 40 immunoglobulin preparations, which were derived from the plasma of healthy individuals who have largely been vaccinated against or exposed to rubella virus in the past. Similar patterns of neutralization were seen for all rubella virus strains (ie, lowest neutralization by US source plasma immunoglobulin lots and significantly higher potency in recovered plasma and EU source plasma immunoglobulin lots). The lower rubella virus nAb titers found in US source plasma, collected primarily from younger donors [13], is likely a reflection of this donor cohort being vaccinated, and MMR vaccination has been shown to induce lower rubella antibody titers compared with earlier rubella virus infection in the advanced-age donor population for recovered plasma [14, 15]. The higher antibody titers found in the immunoglobulin lots produced from plasma collected in the EU, regardless of the modality of plasma collection, and thus, also average age, is likely a result of higher rubella incidence rates in the European region versus the United States in recent decades [16].

The United States introduced the vaccine in 1969 and subsequently maintained a high level of control over virus circulation, leading to virus elimination in 2004. The later onset of rubella vaccination programs across European countries likely resulted in more donors being exposed to natural infection [2]. These results are in line with a previous report [10], in which approximately 1.6-fold higher enzyme-linked immunosorbent assay rubella antibody concentrations were found in immunoglobulin lots manufactured from Czech plasma than in immunoglobulin lots from US plasma.

The generally observed lower nAb titers against iVDRV could be due to antigenic mismatches between the parental vaccine strain and the drifted patient isolates. We found the iVDRV CA isolate least susceptible to neutralization in this study, with amino acid substitutions in 3 of 4 known neutralizing epitopes within the rubella virus E1 protein, compared with more conserved epitopes in isolates OR, RI, and LA [5] (Table 1). The observed differences in nAb titers against the iVDRV isolates were not due to different particle counts used in the neutralization assays, as comparable genomic RNA-to-infectivity ratio were observed in all strains except OR. A higher particle-to-infectivity ratio for the OR isolate versus the respective vaccine virus preparation is unlikely the cause of the observed neutralization differences, as this strain grows to low titers with low expression of viral proteins and produces very small foci [5], indicating a possible underestimation of infectivity by the enzyme-linked immunospot assay used.

All rubella virus isolates were neutralized by immunoglobulin of US and EU origin, consistent with the results generated for 10 serum samples from healthy adult vaccinees against RA27/3 and these 4 iVDRV strains [5]. The smallest difference regarding neutralizing capacity against isolates compared with the vaccine rubella strain was observed for the isolate LA with the least diverged structural proteins (Table 1), which are most likely targets for nAbs. Immunoglobulin preparations had a ≥5-fold lower neutralizing capacity against isolates OR, RI, and CA with more diverged structural proteins. However, even the greatest difference in neutralization that was observed for iVDRV isolate CA (approximately 6-fold) is not sufficient for definition of a new rubella virus serotype. Although the study was limited to a few isolates; considering the possibility that viruses contained in granulomas may be more difficult to neutralize and the limitation that T-cell responses were not considered in this study, our data provide evidence that immunoglobulin preparations, regardless of geographic origin and mode of plasma collection (ie, source or recovered) have potency to neutralize rubella virus strains, more for the vaccine strain and somewhat less for the iVDRV strains. However, there is a trend for more potent neutralization of iVDRV strains by immunoglobulin preparations with increasing titers against the rubella vaccine strain (data not shown).

Overall, these results support the importance of early diagnosis of PID, to commence immunoglobulin substitution and avoid unprotected exposure to rubella virus, either by natural infection or through vaccination. Furthermore, these results suggest that patients with PID receiving immunoglobulin replacement therapy are protected from infection by rubella virus. For treatment of patients with PID and granuloma, for whom standard immunoglobulin administration after rubella infection does not seem to eliminate infection, the use of high-titer immunoglobulin lots (ie, immunoglobulin from recovered plasma), and an increase in immunoglobulin dosage from 0.5 to 2.0 g/kg (recommended dose for, eg, Kawasaki disease) could be a strategy to limit the spread of iVDRV. However, this approach would need evaluation in a clinical setting. These results contribute to a better understanding of the rubella neutralizing ability of immunoglobulin and informs practitioners about the potent neutralizing capacity of immunoglobulin to protect patients for whom rubella live vaccination is contraindicated and to provide a treatment strategy for clinical improvement in patients with already developed rubella virus–associated disease.

## Notes

***Acknowledgments.*** The contributions of Kathrin Nagl (immunoglobulin logistics, Global Pathogen Safety, Takeda Manufacturing Austria AG) and Joël Canivet (lot identification for plasma collection modality, Supply Chain, Takeda Belgium) are gratefully acknowledged.

***Author contributions*.** Study conception and design: L. P., M. R. F., and T. R. K. Data collection: M. C., L. P., L. H., and R. S. B. Analysis and interpretation of results: C. L., M. R. F., M. K., and T. R. K. Draft manuscript preparation: L. P., C. L., M. R. F., M. K., J. I., and T. R. K. All authors reviewed the results and approved the final version of the manuscript.

***Financial support*.** This work was supported by Takeda Manufacturing Austria AG (provision of the immunoglobulin samples tested).
